# Recent advances in mitochondria-targeted porphyrin-based metal-organic frameworks for enhanced cancer therapy

**DOI:** 10.3389/fphar.2026.1764901

**Published:** 2026-01-28

**Authors:** Jiawen Tao, Zhifei Yuan, Mengjiao Zhou

**Affiliations:** 1 The People’s Hospital of Danyang, Affiliated Danyang Hospital of Nantong University, Danyang, Jiangsu, China; 2 School of Pharmacy, Nantong University, Nantong, Jiangsu, China

**Keywords:** metal-organic frameworks, mitochondria, photodynamic therapy, porphyrin, sonodynamic therapy

## Abstract

Porphyrin-based metal-organic frameworks (MOFs) offer exceptional advantages for cancer therapy, including high photosensitizer loading, tunable nanostructures, and suppression of porphyrin self-quenching. By functionalizing with mitochondria targeting ligands, these platforms deliver reactive oxygen species (ROS) precisely to mitochondria, the oxygen-rich and ROS-sensitive organelle, dramatically enhancing photodynamic therapy (PDT) efficacy. This design paradigm has been successfully extended to sonodynamic therapy (SDT) and radiotherapy/radiodynamic therapy (RT-RDT), where porphyrin-MOFs integrate additional functions such as glutathione depletion, CO/H_2_S gas release, or immune activation. Upon ultrasound or X-ray irradiation, these systems synergistically amplify mitochondrial oxidative damage, overcoming hypoxia, antioxidant defenses, and apoptosis resistance. The diversified applications (PDT, SDT and RDT) exemplifies a multimodal strategy that leverages the unique physicochemical properties of porphyrin-MOFs to achieve spatiotemporally controlled, organelle-specific therapy. Looking ahead, the development of intelligent, stimuli-responsive porphyrin-MOF nanoplatforms holds great promise for clinical translation, enabling integrated theranostics and personalized cancer treatment through precise mitochondrial targeting.

## 1 Introduction

Photodynamic therapy (PDT) has garnered significant attention in recent years as a simple, cost-effective, and non-invasive cancer treatment (Pham et al., 2021). PDT utilizes specific wavelengths of light to activate photosensitizers at the tumor site (Xie et al., 2021). This activation generates cytotoxic reactive oxygen species (ROS), particularly singlet oxygen (^1^O_2_), leading to apoptosis or necrosis of cancer cells (Zhao X. et al., 2021; Xie et al., 2024; Zheng et al., 2021). With favorable biocompatibility, porphyrins and their derivatives are widely recognized organic small molecules used in phototherapy (Tian et al., 2020). However, porphyrin-based PDT faces several challenges in clinical applications. These include: (i) The hydrophobic nature of porphyrin molecules, which leads to aggregation in physiological solutions (Zou et al., 2024a). (ii) Hypoxic conditions within solid tumors (Zhang et al., 2020; Dong et al., 2021; Shen et al., 2021). (iii) The short lifespan and limited diffusion radius of ROS (Lee et al., 2021; Sun et al., 2021; Xu X. et al., 2023; Ding et al., 2024). (iv) The maximum absorption wavelength of porphyrin molecules, approximately 650 nm, limits their efficacy in photodynamic therapy against deep-seated tumor cells. The production of ROS is highly dependent on the presence of oxygen (O_2_) (Wan et al., 2021; Wei et al., 2021; Zuo et al., 2024). Thus, PDT efficiency in hypoxic regions is significantly compromised (Zhu et al., 2022a; Tang et al., 2023; Zhang C. et al., 2023). Additionally, the short-lived ROS must act quickly on critical cellular targets to be effective (Dewaele et al., 2010; Murphy et al., 2022).

To overcome these obstacles and achieve optimal PDT outcomes, researchers focus on developing novel carrier materials and post-modification strategies (Zheng et al., 2017; Zhang et al., 2019; Yu et al., 2020). Porphyrin-based nano metal-organic frameworks (Por-nMOFs) have emerged as promising multifunctional platforms in PDT (Chen et al., 2021; Wang et al., 2021; Xu D. et al., 2023; Shano et al., 2024). These materials offer high surface area and tunable pore structures, effectively encapsulating drugs, enzymes, and other bioactive molecules (Wu and Yang, 2017; Zhou et al., 2018; Falsafi et al., 2021; Liu et al., 2021). They also serve as excellent photon energy conversion media (Lu et al., 2014; Lu et al., 2015; Zhang et al., 2024; Qi et al., 2025). The periodic and ordered structure of nMOFs prevents the aggregation of hydrophobic porphyrin molecules, maintaining high ^1^O_2_ quantum yields (Lismont et al., 2017; Zou et al., 2024b; Gong et al., 2025; Zou et al., 2025). Their porous nature facilitates the transport of O_2_ and ROS, enhancing the oxidative damage to cancer cells (He et al., 2019; Wang et al., 2019). Notably, Por-nMOFs exhibit higher drug loading capacity than polymeric nanoparticles and most inorganic nanocarriers, and superior structural stability for multimodal therapy integration compared with covalent organic frameworks (COFs). In terms of therapeutic applicability, Por-nMOFs show better biocompatibility than heavy metal-based inorganic nanocarriers. These properties make nMOFs ideal carriers for photosensitizers and potent photoreactive substances (Zhang et al., 2019). Inside the cell are multiple organelles with distinct functions, which are of vital importance for maintaining the normal metabolic activities of the cell (Handwerger and Gall, 2006; Zimmermann et al., 2024). In the research area of cancer treatment, researchers have discovered that through post-modification to endow various nanomedicines with targeting ability and then make them act on specific organelles, the efficacy of the drugs can be remarkably enhanced unexpectedly (Gao P. et al., 2019). Surprisingly, Por-nMOFs can be modified for targeted delivery to specific tissues or organelles, further improving therapeutic efficacy (Tabish et al., 2023; Jiang X. et al., 2023; Yang et al., 2024).

The effectiveness of cancer therapy depends not only on the total intracellular concentration of a drug but, more critically, on its precise localization within key subcellular structures (Singh et al., 2022; Gong et al., 2024; Iaconis et al., 2024; Chen et al., 2023a; Sun et al., 2024a; Cho et al., 2025; Tang et al., 2025). Organelles, as the basic functional units of the cell, play central roles in maintaining metabolism, energy production, and programmed cell death (Hwang and Jung, 2021; Zhuang et al., 2021; Yang et al., 2022; Wei and Yang, 2023). Therefore, achieving targeted delivery of therapeutic agents to specific organelles holds the promise of generating higher local drug concentrations, thereby eliciting stronger killing effects with lower systemic doses (Ding et al., 2025; Jiang et al., 2025; Liu C. et al., 2025). Among various organelles, mitochondria stand out as highly attractive therapeutic targets due to their unique biological status (Liu P. et al., 2025; Peng et al., 2025; Shan et al., 2025; Teng et al., 2025). Mitochondria are the “powerhouses” of the cell, responsible for adenosine triphosphate (ATP) production *via* oxidative phosphorylation (Wang et al., 2025; Zheng et al., 2025; Zhong et al., 2025; Huang et al., 2026). Notably, oxygen is selectively translocated to the mitochondrial matrix *via* voltage-dependent anion channels and lipid bilayers to support oxidative phosphorylation, thereby mediating localized O_2_ enrichment. In the tumor microenvironment, despite hypoxia induced by disorganized vasculature in rapidly proliferating tumor cells, mitochondrial O_2_ homeostasis is relatively maintained through adaptive metabolic reprogramming, which preserves the efficacy of O_2_-dependent therapeutic modalities such as PDT. Extensive evidence underscores the pivotal role of mitochondria in the pathogenesis of diverse diseases, positioning mitochondrial function as a critical therapeutic target across a spectrum of pathological conditions, including neurodegenerative disorders, metabolic cardiomyopathies, heart failure, neonatal intestinal injury, and cancer (Cheng et al., 2021; Song et al., 2021; Xu et al., 2020; Ya et al., 2020; He-Yang et al., 2020). Moreover, they are also major sites of ROS generation and key regulators of apoptotic pathways. Targeting therapeutic agents to mitochondria offers multiple strategic advantages: Firstly, the mitochondrial membrane potential (negative inside) facilitates the electrostatic accumulation of cationic targeting molecules, enabling highly efficient localization (Alpert et al., 2020; Bazhin et al., 2020; Lin et al., 2021; Gao et al., 2024; Folgar-Cameán et al., 2025). Secondly, direct ROS generation within mitochondria *via* targeted PDT can more effectively induce mitochondrial membrane depolarization, disrupt the electron transport chain, and trigger a cascade of amplified apoptotic signals (Pan et al., 2023; Wang et al., 2023; Dong et al., 2024). Furthermore, targeted intervention in the mitochondrial respiratory chain can directly reduce O_2_ consumption, alleviating tumor hypoxia at its source and creating powerful synergy with O_2_-dependent PDT (Huang et al., 2022; Kadkhoda et al., 2022; Meng et al., 2023; Huang et al., 2025). Thus, developing mitochondrial-targeted Por-nMOFs, aiming to precisely direct the ROS “storm” to the “Achilles’ heel” of tumor cells, is a key strategy for achieving breakthroughs in PDT efficacy.

Despite significant progress in PDT, its reliance on light sources remains a factor limiting its application to tumors in all locations (Sun et al., 2022; Cui et al., 2023; Piksa et al., 2023; Kwon, 2025). To overcome this limitation, the research horizon has expanded from photoexcitation to other physical energy excitation modalities, constructing a more comprehensive “dynamic therapy” system. Sonodynamic therapy (SDT) utilizes ultrasound as the excitation source, offering much greater tissue penetration depth than visible or even near-infrared light, enabling non-invasive treatment of deep-seated tissues (Cao et al., 2023; Jiang Z. et al., 2023; Liu et al., 2023; Song et al., 2023; He F. et al., 2024; Sun et al., 2024b). Ultrasound-induced cavitation is central to SDT, involving microbubble nucleation, expansion and collapse. This process creates extreme local conditions that trigger ROS production. Accumulated ROS induce two complementary cell death pathways: apoptosis, mediated by mitochondrial outer membrane permeabilization which releases pro-apoptotic factors to activate the caspase cascade and culminate in programmed cell death; and ferroptosis, driven by impaired glutathione peroxidase 4 (GPX4) activity that compromises lipid peroxide scavenging, leading to excessive lipid peroxidation production and cell membrane disruption. Additionally, cavitation enhances membrane permeability to facilitate sonosensitizer internalization and ROS diffusion. Porphyrin MOFs as ultrasound-activated sonosensitizers can similarly generate cytotoxic ROS, and ultrasound itself may further enhance cell membrane permeability and drug internalization through mechanisms like cavitation effects.

Radiodynamic therapy (RDT) represents another promising direction, combining radiotherapy with dynamic therapy (Huang et al., 2023; Kirakci et al., 2023; Li et al., 2023a). Its principle involves irradiating nanosensitizers containing high atomic number elements with high-energy ionizing radiation (X-rays) (Li et al., 2023b; Li T. et al., 2023; Xiong et al., 2024). The radio-sensitization and RDT-mediated therapeutic mechanisms are multi-faceted: high-Z elements efficiently capture X-ray photons and undergo photoelectric effect to generate high-energy Auger electrons and photoelectrons. These electrons directly induce lethal, repair-resistant DNA double-strand breaks and trigger water radiolysis to produce free radicals (·OH), exacerbating oxidative DNA damage such as base oxidation and single-strand breaks. Concurrently, absorbed energy transfers to porphyrin ligands in MOFs, activating them to generate ^1^O_2_ that attacks intracellular biomacromolecules like proteins and lipids. The synergy of high-Z element-mediated direct DNA damage and dynamic therapy-derived ROS oxidative damage enhances tumor cell killing, especially in radioresistant tumors with overactivated DNA repair machinery (Lan et al., 2018; Xu et al., 2022; Zhao et al., 2023; Lin et al., 2024; Zhen et al., 2024; Du et al., 2025). The evolution from PDT to SDT and RDT, from light to sound to ionizing radiation, essentially represents an adaptive breakthrough against the varying depths and physiological barriers of tumor tissues. Combining porphyrin MOFs with these modalities allows for the construction of versatile therapeutic platforms without being limited by light penetration depth and capable of exploiting tumor microenvironment characteristics, such as overexpressed hydrogen peroxide (H_2_O_2_), greatly expanding their application scope and therapeutic potential.

**FIGURE 1:**
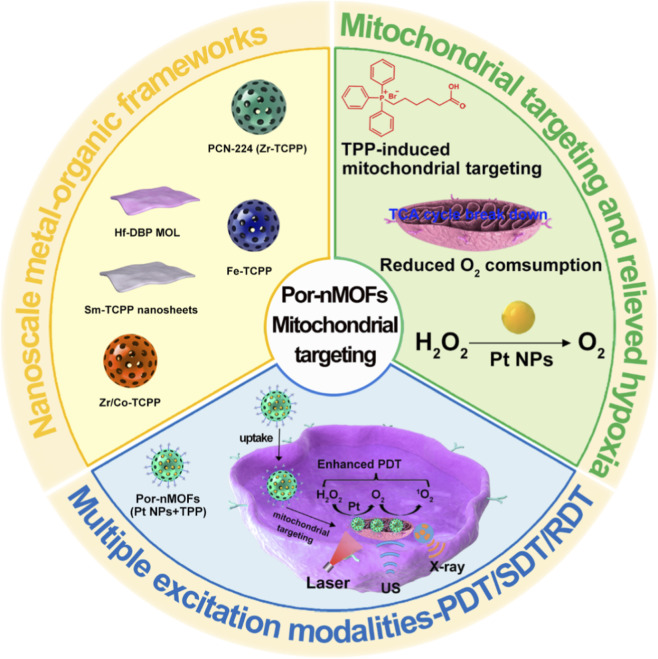
Preparation of various Por-nMOFs, their mechanisms for targeting mitochondria and alleviating hypoxia, and application in enhanced PDT/SDT/RDT.

Given the structural and functional diversity of Por-nMOFs, the strategic significance of mitochondrial targeting in subcellular therapy, and the complementary advantages of multiple energy excitation modalities in overcoming tumor physiological barriers, the integrated combination of these three aspects has become a highly promising frontier in nanoscale oncology (Figure 1). Distinct from existing reviews that focus on either general MOF platforms or single-modal PDT/SDT, this work centers on the synergistic integration of Por-nMOF materials, mitochondrial subcellular targeting, and the progressive evolution from PDT to SDT and further to RDT. We specifically highlight two unique innovations: first, the in-depth dissection of the structure-activity relationship between Por-nMOF design and mitochondrial targeting efficiency; second, the systematic comparison of how different energy sources synergize with mitochondrial targeting to amplify ROS-mediated cell death. This review outlines the traditional challenges of porphyrin-based PDT and the unique value of MOFs as a solution platform, then elaborates on mitochondrial targeting strategies and their core mechanisms in amplifying ROS-mediated cell killing. Subsequently, it discusses representative Por-nMOF-based systems, with dedicated focus on hypoxia-alleviating designs for PDT, cavitation-driven ROS amplification mechanisms for SDT, and heavy metal radiosensitization combined with gas therapy synergies for RDT. Ultimately, this review reveals the design principles of “material-targeting-energy” synergy, explores trends in intelligent theranostic platforms, and provides a theoretical reference for precise tumor therapy.

## 2 Advantages of MOFs as porphyrin carriers in cancer therapy

Compared to traditional nanocarriers such as liposomes and polymer nanoparticles, Por-nMOFs exhibit multiple unique advantages as photosensitizer carriers. Structurally, MOFs are formed by the periodic assembly of metal nodes and organic linkers, providing high-density, predictable, and spatially isolated sites for porphyrin photosensitizers (Gao Z. et al., 2019; Xu D. et al., 2023; Ouyang et al., 2025; Zhang J. et al., 2025). This not only achieves an exceptionally high drug loading capacity but, more importantly, effectively prevents π-π stacking and self-quenching of porphyrin molecules, thereby significantly enhancing the ^1^O_2_ generation efficiency (Pan et al., 2022; Zhang et al., 2022; Zhang J. et al., 2023). Their highly ordered nanoporous structures also facilitate the rapid diffusion of O_2_ and ROS. Compared to COFs, MOF synthesis is generally more straightforward, and their metal nodes (*e.g.*, Zr^4+^, Hf^4+^, Fe^3+^) can inherently confer additional therapeutic functionalities. For instance, the strong X-ray attenuation ability of Hf clusters can be utilized for radiosensitization, while transition metal ions like Fe^3+^ can catalyze Fenton-like reactions for chemodynamic therapy (CDT).

In tumor therapy, the uniqueness of Por-nMOFs lies in their exceptional potential for multimodal synergy and precise regulation. In PDT, MOFs can act directly as “fourth-generation photosensitizers,” synergistically overcoming tumor hypoxia by targeting mitochondria and integrating O_2_-generating nanozymes. In SDT and RDT, the rigid framework and tunable metal composition of MOFs enable efficient absorption of ultrasound or X-ray energy and effective energy transfer to porphyrin linkers to produce ROS. Simultaneously, by loading gas prodrugs or immune adjuvants, gas therapy or immunomodulatory synergistic therapy can be achieved (Cao et al., 2025). This ability to integrate diagnosis, targeting, multiple treatment modalities, and microenvironment regulation into a single, stable platform is difficult to match with traditional carriers or most current COF platforms, offering more potential for constructing intelligent and efficient theranostic systems for tumors. To systematically illustrate the design principles, therapeutic performance, and *in vivo* outcomes of these versatile Por-nMOF platforms, a comprehensive summary is presented (Table 1).

**TABLE 1:** Mitochondria-targeted porphyrin-based MOFs against cancer.

Materials and targeting	Therapy mode	Tumor model and Regimen	Therapeutic efficacy	Advantages and limitations	References
UiO-66-TCPP; TPP, FA	PDT	SMMC-7721 cancer cells; 650 nm light,10 min	^1^O_2_ yield ∼12.5; IC_50_ ∼0.74 μM	Advantages: both cancer cell- and mitochondria-targeting; Limitations: complex synthesis	Gong et al. (2020)
PCDTs; TPP	PDT	MCF-7 breast cancer; 808 nm laser, 10 min (1.0 W cm^−2^, 3 min interval for every 1 min)	Cell viability ∼15% (100 μg mL^−1^); tumor accumulation ∼4.7-folds higher than that in the liver	Advantages: absorb NIR laser to transfer energy to Por-nMOF; Limitations: complex synthesis	Xiang et al. (2022)
TPP-UCNPs@MOF-Pt; TPP	PDT	HeLa cervical cancer; 980 nm laser, 10 min, 1.5 W cm^−2^	Loading PtNPs ∼17.8 wt%; low cytotoxicity (200 μg/mL); 100% survival rate in 60 days	Advantages: dual hypoxia-alleviating capability; Limitations: complex synthesis, potential biocompatibility risks	Chen et al. (2023b)
7ACC2/DOX@PCN-224@ZIF-8 (AD@PZ); no targeting	PDT + chemotherapy	SiHa cervical cancer; 660 nm laser, 5 min, 200 mW cm^−2^	Negligible hemolytic activity (150 μg/mL); tumor growth inhibition ∼70%	Advantages: alleviate tumor hypoxia, prevents drug leakage; Limitations: lack active targeting capability	Yu et al. (2023)
HA@MR@PCN-CORM; HA, TPP	PDT + gas therapy + ferroptosis	4T1 breast cancer; 660 nm laser, 5 min, 1 W cm^−2^	IC_50_ ∼0.23 μg/mL; CI < 1 revealed synergism; tumor growth inhibition ∼90%	Advantages: multimodal antitumor effect, dual-targeting capability, ROS-responsive release; Limitations: complex synthesis, potential biocompatibility risks	Yang et al. (2023)
BSO-TCPP/Fe@CaCO_3_-PEG; no targeting	SDT + mitochondria damage + GSH depletion	4T1 breast cancer; ultrasound (40 kHz, 10 W, 60 min)	Intracellular GSH content ∼49.2%; tumor accumulation ∼9.9% ID/g; tumor growth inhibition ∼79.6%	Advantages: tumor-responsive release, mitochondrial dysfunction (Ca^2+^) and GSH depletion (BSO) amplifies oxidative stress; Limitations: lack targeting capability	Dong et al. (2020)
Zr-TCPP(TPP)/R837@M; TPP, cancer cell membrane	SDT + immune therapy	4T1 breast cancer; ultrasound (3 MHz, 1.5 W cm^−2^, duty cycle 50%)	Eradicate primary 4T1 tumors ∼59.5%, suppressing distant tumor growth ∼61.4%, 40% survival rate in 50 days	Advantages: dual-targeting capability, improved biocompatibility, synergistic therapy; Limitations: potential off-target toxicity risks	Luo et al. (2022)
SHF@PMOF; no targeting	RDT + CO/H_2_S gas therapy	4T1 breast cancer; 6 Gy dose of X-ray radiation	Low cytotoxicity (400 μg/mL); half-life (t_1/2_) ∼4.17 h; no remarkable inflammatory lesions or tissue damage	Advantages: deep-tissue penetration, multi-modal synergistic antitumor effect; Limitations: dual-gas release control and off-target risks	Cao et al. (2025)

## 3 Mitochondria-targeted delivery for enhanced PDT

### 3.1 Design and optimization of targeting strategies

**FIGURE 2:**
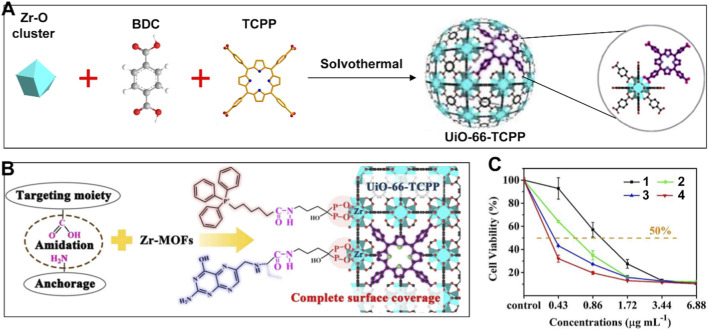
**(A)** Synthesis procedure for UiO-66-TCPP. **(B)** Targeted modification of UiO-66-TCPP. **(C)** Cytotoxicity to SMMC-7721 cancer cells after different treatments: (1) UiO-66-TCPP, (2) UiO-66-TPP, (3) UiO-66-FA, and (4) UiO-66-TPP-FA. Adapted with permission from Gong et al. (2020). © 2019 Wiley-VCH Verlag GmbH & Co. KGaA, Weinheim.

As the powerhouses of the cell, mitochondria are responsible for generating energy through a series of complex biochemical reactions known as aerobic respiration (Harrington et al., 2023; Qian et al., 2024). For mitochondrial respiration, O_2_ is transported from the extracellular environment to the cytoplasm *via* hemoglobin, then traverses the mitochondrial outer and inner membranes. The inner membrane’s lipid bilayer and specific channels are responsible for facilitating the selective entry of O_2_ into the mitochondrial matrix. In this process, O_2_ serves as the final electron acceptor in the electron transport chain, facilitating the release of high-energy electrons from nutrients and their conversion into a substantial amount of adenosine triphosphate, which is essential for cellular functions (Borcherding and Brestoff, 2023). Consequently, mitochondria are one of the most O_2_-rich organelles within the cell (Peng et al., 2023). Since the Type II PDT mechanism of most photosensitizers relies on the presence of O_2_, designing Por-nMOFs that target mitochondria represents an effective strategy to mitigate the limitations imposed by hypoxia. For instance, Gu et al. developed a one-pot synthesis method to incorporate tetrakis (4-carboxyphenyl)porphine (TCPP) into UiO-66, creating a photoactive platform (Figure 2A) (Gong et al., 2020). To alleviate the constraints of hypoxia on the ^1^O_2_ generation capability of the photosensitizer and to achieve enhanced phototherapeutic efficacy, the authors leveraged the facile surface modification properties of MOFs. This allowed them to functionalize UiO-66-TCPP with both cancer cell-targeting and mitochondria-targeting capabilities. Specifically, they first synthesized phosphonated triphenylphosphonium (TPP) and folic acid (FA) *via* amide reactions. The phosphate groups were then used to form stable Zr-O-P bonds with the Zr nodes in the UiO-66 framework, thereby achieving the surface functionalization with FA and TPP (Figure 2B). This system ingeniously utilized the structural characteristics of MOFs, which are formed through the coordination of metal clusters with carboxylic acid ligands, to integrate targeting functionalities. Cytotoxicity assays demonstrated that both FA-induced cancer cell targeting and TPP-induced mitochondrial targeting significantly enhanced the phototoxicity of UiO-66-TCPP. The IC_50_ value of the dual-targeted Por-nMOFs was as low as 0.74 μM, which is four times lower than that of the non-targeted control group (Figure 2C). This study provides a valuable design strategy for mitochondria-targeted Por-nMOFs and effectively demonstrates that mitochondrial targeting may offer new opportunities for the phototherapeutic applications of these materials.

**FIGURE 3:**
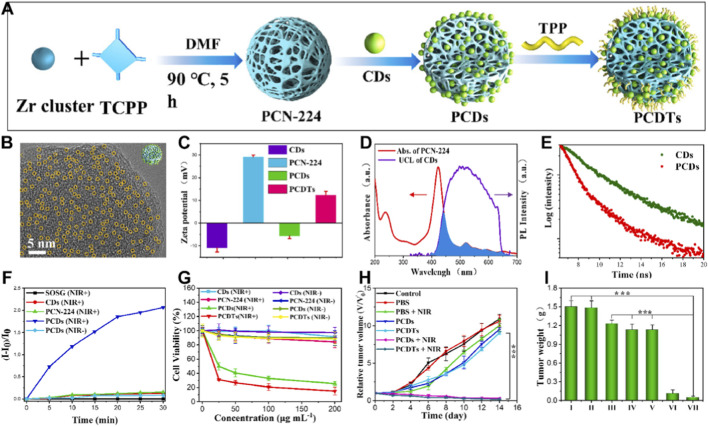
**(A)** Synthetic procedures of PCDs and PCDTs. **(B)** HRTEM images of PCDs. **(C)** Zeta potentials of CDs, PCN-224, PCDs and PCDTs at same conditions. **(D)** The UV-vis absorption spectrum of PCN-224 and the UCL spectrum of CDs are presented, with the overlapping part shown in blue. **(E)** UCL decay curves of CDs and PCDs are shown. **(F)** The generation of ^1^O_2_ triggered by NIR light in CDs, PCN-224, PCDs and PCDTs was determined through the SOSG assay. **(G)** Control experiments of cytotoxicity in MCF-7 cells for 24 h at different concentrations, either with or without NIR light irradiation, were conducted. **(H)** The relative tumor volume and **(I)** the tumor weight of tumor-bearing mice under different treatments were measured. Adapted with permission from Xiang et al. (2022). © 2022 Elsevier B.V.

In addition to covalently grafting TPP and folic acid (FA) onto the surface of Zr-nMOFs to achieve dual targeting of cancer cells and mitochondria, researchers have developed a near-infrared (NIR)-light-triggered heterostructure comprising upconversion carbon dots integrated with Por-nMOFs. This system also employs TPP for mitochondrial targeting. Upon NIR irradiation, efficient energy transfer through tight coupling significantly enhances ^1^O_2_ generation, while TPP-mediated mitochondrial localization amplifies oxidative damage. For example, Zhang et al. synthesized PCN-224 MOF using a conventional solvothermal method (Xiang et al., 2022). They then sonicated, stirred, and freeze-dried the MOF with carbon dots (CDs) prepared *via* a hydrothermal method using L-ascorbic acid as the carbon source, resulting in the PCDs material. By further stirring and freeze-drying the PCDs with TPP solution, they obtained the TPP-modified PCDTs nanoplatform (Figure 3A). Transmission electron microscope (TEM) images showed that 2 nm-sized CDs were uniformly distributed within the PCN-224 MOF (Figure 3B). The O_2_-rich groups on the surface of the CDs imparted a negative surface potential, which neutralized the positive charge of the PCN-224 MOF, making the composite’s potential negative. However, the positive charge introduced by TPP modification restored the composite’s positive potential, as expected (Figure 3C). To demonstrate the effectiveness of CDs as a light energy conversion medium, the authors measured the absorption spectrum of PCN-224 and the emission spectrum of CDs, which showed significant overlap. This result indicated that CDs could effectively achieve fluorescence resonance energy transfer (FRET) to PCN-224 (Figure 3D). Time-resolved photoluminescence (PL) experiments revealed a significant shortening of the PL lifetime of PCDs at 520 nm (Figure 3E), further confirming the energy transfer from CDs to PCN-224. Under 808 nm laser irradiation, the use of singlet oxygen sensor green (SOSG) as a ^1^O_2_ scavenger showed that PCDs could effectively generate ^1^O_2_, whereas direct 808 nm laser irradiation of PCN-224 did not produce ^1^O_2_ (Figure 3F). This result further validated the FRET mechanism in the PCDs structure. Cell viability assays using the CCK-8 method demonstrated that both PCDs and PCDTs exhibited excellent biocompatibility under non-irradiated conditions. However, under 808 nm laser irradiation, they effectively inhibited cancer cell proliferation. With the advantage of targeting mitochondria, PCDTs are highly sensitive to ROS, showed the best inhibitory effect on cancer cell proliferation (Figure 3G).

*In vivo* animal experiments also confirmed that PCDs and PCDTs achieved satisfactory tumor volume inhibition under 808 nm laser irradiation (Figure 3H). The tumor weight comparison results were consistent with expectations (Figure 3I). Moreover, even in intratumoral experiments, mitochondrial targeting demonstrated superior antitumor effects. This system leverages the natural porous structure and facile surface modification of Por-nMOFs to achieve a tight integration of well-dispersed, upconverting CDs within the PCN-224 MOF framework. The O_2_-rich groups on the CDs and the unsaturated metal sites in the MOF form a stable heterojunction structure, which reduces the distance between the upconversion material and the photosensitizer, facilitating more efficient energy transfer. Both *in vitro* and *in vivo* experiments demonstrated the effective FRET process within this framework. Furthermore, the TPP modification endowed the nanoplatform with mitochondrial-targeting properties, and by amplifying oxidative stress within the mitochondria, it achieved enhanced PDT efficacy.

### 3.2 Alleviating tumor hypoxia to synergistically potentiate PDT

**FIGURE 4:**
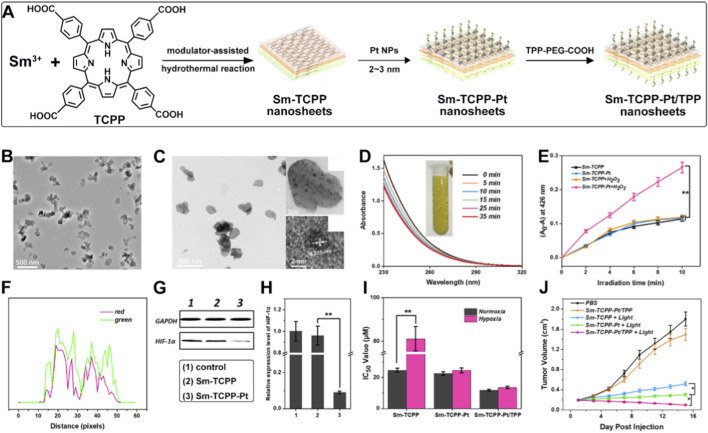
**(A)** Preparation procedures of Sm-TCPP-Pt/TPP. **(B)** TEM images of Sm-TCPP and **(C)** SmTCPP-Pt. **(D)** Changes of the degradation curve of H_2_O_2_ effected by Sm-TCPP-Pt. **(E)** ^1^O_2_ generation efficiency of Sm-TCPP and Sm-TCPP-Pt in the presence or absence of H_2_O_2_ under hypoxia conditions (**p < 0.01). **(F)** Fluorescence co-localization of red light from the mitochondrial staining kit and green light from the labeled sample. **(G)** Western blot analysis of HIF-1α expression in MCF-7 cells and **(H)** quantitative analysis using ImageJ software: (1) control, (2) Sm-TCPP, (3) Sm-TCPP-Pt. **(I)** IC50 values after different treatments. **(J)** Tumor volume changes after various treatments. Adapted with permission from Gao et al. (2020). © 2019 American Chemical Society.

Direct functionalization of Por-nMOFs with mitochondrial-targeting ligands such as TPP is an effective and widely adopted strategy to enhance PDT efficacy. However, these studies also indicate that merely increasing mitochondrial accumulation may be insufficient to overcome the complex challenges posed by the tumor microenvironment, particularly the pervasive hypoxia, which highlights a clear direction for subsequent strategic advancements (Li et al., 2018; Lu et al., 2019; Huang et al., 2021). The hypoxic tumor microenvironment severely compromises the efficiency of Type II PDT (Liang et al., 2023; He and Ma, 2024; Xia et al., 2024). Consequently, researchers have increasingly focused on integrating mitochondrial targeting with “self-oxygenation” or “O_2_-supply-enhancing” capabilities. By endowing MOF-based nanoplatforms with the ability to catalytically decompose endogenous H_2_O_2_ to generate O_2_, or to modulate cellular metabolism to reduce O_2_ consumption, hypoxia can be alleviated at its source, thereby maximizing the therapeutic potential of mitochondria-targeted PDT. For example, Sun et al. synthesized Sm-TCPP nanosheets *via* a conventional solvothermal reaction, coordinating TCPP photosensitizer molecules with Sm^3+^ ions (Gao et al., 2020). They then grew Pt nanoparticles *in situ* on the surface of the Sm-TCPP nanosheets, resulting in the Sm-TCPP-Pt nanosheet material. By leveraging the strong coordination between Sm^3+^ and carboxyl groups, they introduced TPP-PEG-COOH molecules into the Sm-TCPP-Pt platform, yielding a well-dispersed and biocompatible Sm-TCPP-Pt/TPP nanoplatform (Figure 4A). In this system, the Pt NPs function as nanozymes, mimicking the activity of catalase. By catalyzing the decomposition of H_2_O_2_ into O_2_, they increase the intracellular O_2_ concentration, thereby enhancing the PDT efficacy of the photosensitizer. TEM images clearly show the 2D nanosheet morphology of Sm-TCPP (Figure 4B). This 2D structure is more favorable for the photosensitizer to interact with O_2_ molecules and generate ^1^O_2_ under light exposure, potentially offering advantages over 3D MOF-based photosensitizers. Additionally, TEM images of Sm-TCPP-Pt confirm the presence of Pt NPs (Figure 4C), and the results indicate that the *in situ* growth of Pt has a negligible effect on the particle size of Sm-TCPP. To demonstrate the catalase-like activity of the Pt NPs, the authors mixed Sm-TCPP-Pt with H_2_O_2_ and monitored the absorbance at 240 nm to detect changes in H_2_O_2_ concentration. The results showed that over time, more bubbles were produced, and the H_2_O_2_ concentration continuously decreased, confirming the catalytic performance of the Pt NPs (Figure 4D). Next, the authors used 1,3-diphenylisobenzofuran (DPBF) as a ^1^O_2_ scavenger to assess the ^1^O_2_ generation capability of the photosensitizer by monitoring the absorbance changes at 426 nm. Under hypoxic conditions, Sm-TCPP-Pt exhibited significantly enhanced ^1^O_2_ generation compared to Sm-TCPP alone (Figure 4E). This result indicates that the Pt NPs effectively mitigate the limitations imposed by hypoxia on the PDT efficiency of Por-nMOFs. Mitochondrial co-localization experiments demonstrated that FITC-labeled Sm-TCPP-Pt/TPP showed effective fluorescence overlap with a red mitochondrial marker, indicating that TPP successfully mediates the targeting of the Sm-TCPP-Pt/TPP nanoplatform to mitochondria (Figure 4F). At the cellular level, the authors showed that the presence of Pt NPs significantly reduced the levels of hypoxia-inducible factors, indicating their ability to improve the hypoxic environment (Figures 4G,H). Cytotoxicity assays further confirmed that Pt NPs significantly enhanced the cytotoxicity of Sm-TCPP under hypoxic conditions, resulting in a lower IC_50_ value. Moreover, the mitochondrial targeting modification with TPP further increased the toxicity of the Sm-TCPP photosensitizer, as mitochondria are the primary organelles for H_2_O_2_ production, facilitating the generation of more O_2_ by Pt NPs (Figure 4I). The authors then evaluated the *in vivo* inhibitory effects of the Sm-TCPP-Pt/TPP nanoplatform on MCF-7 breast cancer cells. The results showed that the Sm-TCPP-Pt/TPP nanoplatform, with the assistance of Pt nanozymes and TPP-mediated mitochondrial targeting, exhibited the most potent inhibition of cancer cell proliferation (Figure 4J). This system not only targets mitochondria using TPP but also utilizes the catalase-mimetic activity of Pt NPs to decompose H_2_O_2_ and generate O_2_, thereby alleviating the hypoxic environment. Under light exposure, this combination effectively suppresses the growth of MCF-7 cancer cells, demonstrating the promising new application prospects of Por-nMOFs in cancer therapy.

**FIGURE 5:**
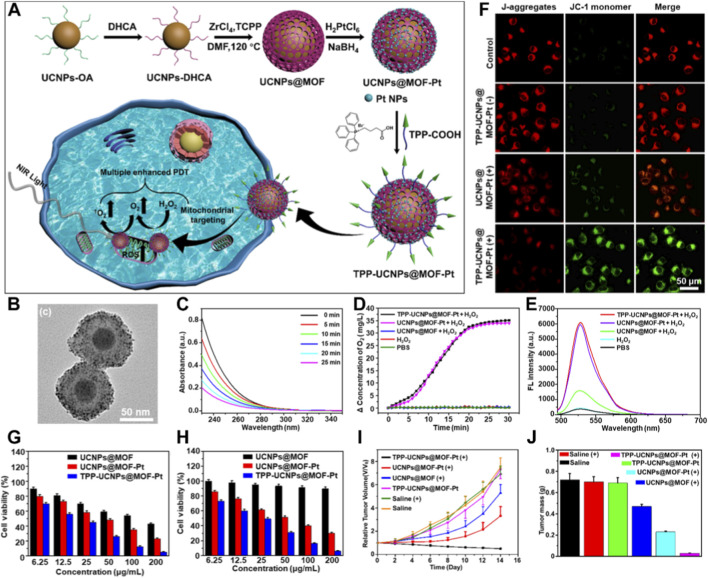
**(A)** Preparation procedures of TPP-UCNPs@MOF-Pt nanoplatform for enhanced PDT. **(B)** Transmission electron microscopy images of UCNPs@MOF-Pt. **(C)** The UV-vis spectra change of H_2_O_2_ after addition of TPP-UCNPs@MOF-Pt. **(D)** O_2_ generation curves after various treatments. **(E)** ^1^O_2_ generation efficiency after various treatments under laser irradiation (980 nm, 1.5 W/cm^2^, SOSG probe). **(F)** The changes of mitochondrial membrane potential of HeLa cells after various treatments. Cytotoxicity of various treatments in normoxic **(G)** and hypoxic **(H)** environment under laser irradiation. **(I)** Volume changes after various treatments. **(J)** Tumor mass results from different treatment groups. Adapted with permission from Chen et al. (2023a). © 2022 Wiley-VCH GmbH.

*In situ* O_2_ generation *via* nanozymes represents one of the effective strategies to alleviate tumor hypoxia (Liu et al., 2022; Nan et al., 2022; Xiao et al., 2022; Zhu et al., 2022b). Similarly, integrating O_2_-generating functionality into more sophisticated core-shell architectures can further enhance the stability and multifunctionality of nanotherapeutic platforms. For instance, Zhu et al. synthesized oleic acid (OA)-modified upconversion nanoparticles (UCNPs-OA) using conventional methods (Chen et al., 2023b). They then performed ligand exchange to obtain 3,4-dihydroxycinnamic acid (DHCA)-modified UCNPs-DHCA. By mixing these with Zr^4+^ ions and TCPP molecules and heating, they prepared a core-shell structured UCNP@MOF material. Subsequently, the UCNP@MOF was mixed with H_2_PtCl_6_ in an ethanol solution, followed by the addition of NaBH_4_, resulting in the formation of UCNP@MOF-Pt nanocomposites. Finally, through the coordination of Zr^4+^ ions with TPP-COOH, they obtained a mitochondria-targeting TPP-UCNP@MOF-Pt nanoplatform, which can perform PDT under 980 nm laser irradiation (Figure 5A). TEM results confirmed the core-shell structure of UCNP@MOF-Pt, with clearly visible 2 nm Pt NPs (Figure 5B). As shown in Figure 5C, the absorbance of H_2_O_2_ at 240 nm decreased over time, indicating a gradual reduction in H_2_O_2_ concentration. This result confirms the catalytic function of Pt NPs in decomposing H_2_O_2_ to produce O_2_. The O_2_ generation curve also verified the catalytic effect of Pt NPs on H_2_O_2_ (Figure 5D). ^1^O_2_ phosphorescence (SOP) measurements showed that the self-generated O_2_ from the nanoplatform effectively enhanced ^1^O_2_ production (Figure 5E).

Next, the mitochondrial membrane potential was assessed. The green fluorescence of JC-1 monomers indicated that the mitochondria-targeting TPP-UCNP@MOF-Pt nanoplatform most effectively reduced the mitochondrial membrane potential, causing mitochondrial depolarization (Figure 5F). Cytotoxicity assays demonstrated that, under normoxic conditions, both mitochondrial targeting and the self-O_2_-generating capability significantly enhanced PDT efficacy (Figure 5G). Under hypoxic conditions, the advantages of the designed nanoplatform were even more pronounced. The results showed that while UCNP@MOF had weaker PDT efficacy, UCNP@MOF-Pt and TPP-UCNP@MOF-Pt, due to their O_2_-generating capabilities, could effectively inhibit cancer cells in a hypoxic environment (Figure 5H). *In vivo* tumor suppression experiments also aligned with these findings. The TPP-UCNP@MOF-Pt nanoplatform, under 980 nm laser irradiation, achieved the best inhibition of tumor volume (Figure 5I) and weight (Figure 5J). This system, through effective chemical synthesis and coordination, produced a core-shell structured UCNP@MOF composite material. It not only leveraged the advantages of NIR light excitation for porphyrin-based PDT but also utilized its intrinsic O_2_-generating function and mitochondrial targeting to provide a novel approach for treating hypoxic tumors.

**FIGURE 6:**
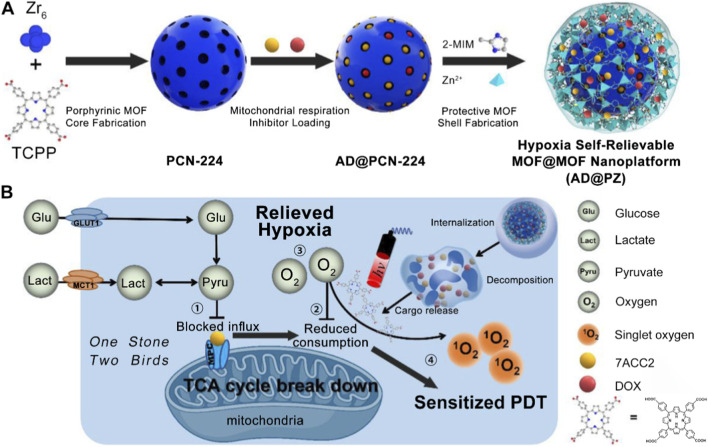
**(A)** Preparation procedure for the MOF@MOF nanoplatform. **(B)** Enhanced PDT mechanism through the inhibition of mitochondrial respiration. Adapted with permission from Yu et al. (2023). © 2023 Acta Materialia Inc. Published by Elsevier Ltd.

In addition to using exogenous nanotherapeutics to catalytically generate O_2_ and ameliorate the hypoxic microenvironment, modulating intracellular metabolic pathways to reduce O_2_ consumption represents another ingenious “O_2_-conserving” strategy. For example, Tian et al. first synthesized PCN-224, a MOF formed by the coordination of Zr^4+^ with TCPP (Yu et al., 2023). Subsequently, 7ACC2 and doxorubicin (DOX) were physically adsorbed into the pores of PCN-224, forming the 7ACC2/DOX@PCN-224 (AD@P) system. To enhance the stability and drug loading efficiency, a layer of ZIF-8 crystal shell was then grown on the surface of AD@P, resulting in the 7ACC2/DOX@PCN-224@ZIF-8 (AD@PZ) platform (Figure 6A). The authors simplified the synthesis steps and improved drug loading by not removing the free DOX and 7ACC2 from the supernatant before the formation of the ZIF-8 shell, which effectively enhanced the encapsulation of small-molecule drugs. The resulting AD@PZ nanoplatform significantly improved the circulation stability of the drug-loaded PCN-224. Upon reaching the cancer cells, the ZIF-8 shell rapidly degraded under weakly acidic conditions, allowing the controlled release of DOX and 7ACC2. The 2-methylimidazole component of ZIF-8 facilitated lysosomal escape *via* protonation, enhancing the intracellular delivery of the drugs. As a mitochondrial pyruvate carrier inhibitor, once the 7ACC2 molecule was released, the metabolic activities of the mitochondria will be affected. The released inhibitor not only inhibited the influx of pyruvate into the mitochondria but also effectively blocked the tricarboxylic acid (TCA) cycle, which is fueled by glucose and lactate, thereby disrupting aerobic respiration and alleviating tumor hypoxia (Figure 6B). By targeting the mitochondria, 7ACC2 successfully reduced O_2_ consumption within the cells, providing a solid foundation for enhanced PDT under light exposure. Ultimately, this system achieved a synergistic therapeutic effect by combining enhanced PDT induced by the inhibition of mitochondrial respiration and chemotherapy mediated by DOX. The design of the dual-layer MOF@MOF structure in this system offers a new reference for improving the circulation stability of Por-nMOFs, contributing to their preclinical development and application.

### 3.3 Beyond apoptosis: Co-induction of novel cell death mechanisms

Integrating catalytic O_2_-generating or metabolism-modulating modules to improve tumor oxygenation has emerged as a pivotal strategy for enhancing the efficacy of mitochondria-targeted PDT. These designs substantially boost ROS generation. Nevertheless, the robust antioxidant defense systems and potential apoptosis resistance mechanisms of tumor cells can still limit the ultimate therapeutic outcomes of PDT (Cheng et al., 2022; Deng et al., 2022; Ren et al., 2025; Wu et al., 2025). These factors prompted researchers to explore synergistic cell-killing pathways beyond conventional apoptosis. To overcome tumor cell tolerance to PDT, recent efforts have focused on combining mitochondria-targeted PDT with mechanisms that induce non-apoptotic forms of cell death. By co-localizing at mitochondria and simultaneously triggering intense oxidative stress, disrupting key antioxidant systems, or releasing specific cytotoxic agents, such strategies can activate more potent cell death programs, such as ferroptosis, enabling efficient eradication of refractory tumors. Ferroptosis, an iron-dependent form of regulated cell death driven by lipid peroxidation, hinges on core redox regulators such as the glutathione (GSH)/glutathione GPX4 axis, making it a compelling therapeutic target across diverse cancer contexts (Ji et al., 2022; Wang et al., 2022; Mao et al., 2023). Mechanistically, ferroptosis is induced by dual perturbations: GSH/GPX4 axis inhibition blocks lipid peroxide detoxification, and dysregulated Fe^2+^ fuels ROS generation *via* Fenton reactions. The resultant excessive lipid peroxidation disrupts membrane homeostasis and initiates ferroptotic cell death. Recent studies collectively demonstrate that modulating iron homeostasis, suppressing antioxidant defenses, or amplifying lipid peroxidation can effectively trigger ferroptotic cell death in tumors, thereby inhibiting progression and overcoming therapy resistance (He J. et al., 2024; Sun Y. et al., 2024; Guan et al., 2025).

**FIGURE 7:**
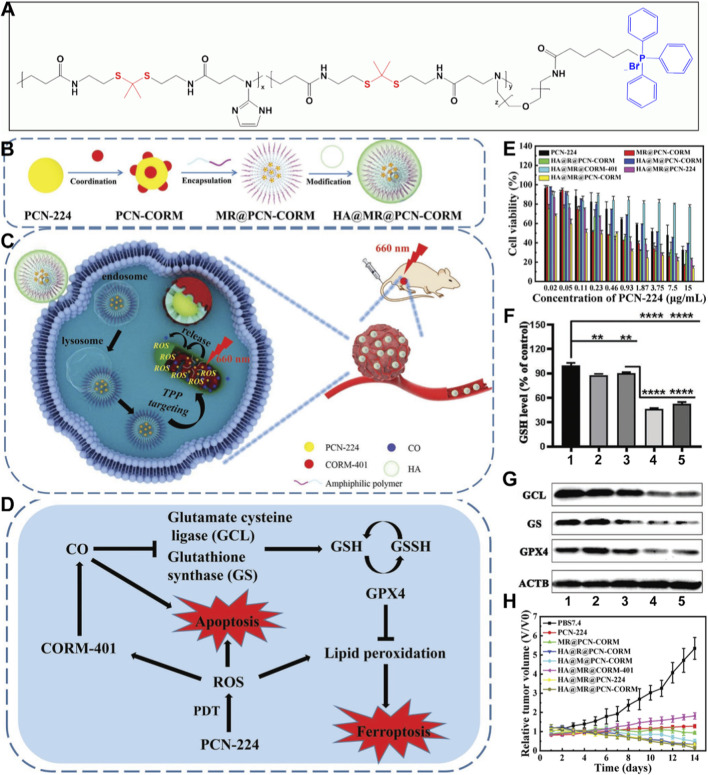
**(A)** The structure of amphiphilic copolymer (TK + TPP groups (MR-AP)). **(B)** Preparation procedure, **(C)** antitumor process, and **(D)** synergistic mechanism of HA@MR@PCN-CORM for combined PDT and gas therapy. **(E)** Cytotoxicity of various treatments. **(F)** GSH levels of various groups. (1) blank, (2) HA@MR@PCN-224, (3) HA@MR@CORM-401, (4) HA@MR@PCN-CORM and (4) positive (**p < 0.01, ****p < 0.0001). **(G)** WB detection of the protein expression levels of GPX4, GS, and GCL in 4T1 cells. **(H)** Tumor volume changes after various treatments (660 nm, 1 W cm^−2^, 5 min). Adapted with permission from Yang et al. (2023). © 2023 Wiley-VCH GmbH.

For instance, Peng et al. developed a mitochondria-targeted nanosystem for the co-delivery of a porphyrin-based MOF (PCN-224) and a carbon monoxide-releasing molecule (CORM). The generated ROS and released carbon monoxide (CO) act synergistically to not only promote apoptosis but also sensitize cancer cells to ferroptosis. Firstly, They synthesized an amphiphilic polymer (MR) (Figure 7A) (Yang et al., 2023). This polymer features a mechanism for cleavage in response to ^1^O_2_ and is functionalized with TPP at its terminus, endowing it with the ability to target mitochondria. These authors then prepared PCN-224 MOFs using a conventional solvothermal method and loaded them with a CO-releasing molecule (CORM) to form PCN-CORM. The resulting PCN-CORM was encapsulated with the MR polymer, yielding MR@PCN-CORM. To mitigate the potential negative impact of the positive charge of TPP on the circulation stability of the nanoplatform, the surface was further modified with HA, resulting in the HA@MR@PCN-CORM nanoplatform (Figure 7B). The prepared HA@MR@PCN-CORM nanoplatform can effectively generate ROS under light irradiation and activate the release of CO, inducing ferroptosis and ultimately achieving enhanced antitumor effects both *in vitro* and *in vivo* (Figure 7C). The mechanism involves the light-induced generation of ROS by PCN-224, which can directly induce apoptosis and stimulate the release of CO from CORM-401. The released CO was found to directly affect the biological activity of glutamate-cysteine ligase (GCL) and glutathione synthetase (GS), leading to a reduction in GSH levels, lipid peroxidation, and the induction of ferroptosis (Figure 7D). The authors confirmed the inhibitory effect of the HA@MR@PCN-CORM nanoplatform on 4T1 cancer cell proliferation using the MTT assay (Figure 7E). The MTT assay is a colorimetric method that measures the activity of enzymes in living cells to evaluate cell viability (Wu et al., 2019; Chen et al., 2020; Hu et al., 2020; Yang et al., 2021; Zhao Y. et al., 2021). The measured IC_50_ value was 0.23 μg/mL, the lowest among all control groups, indicating the highest cytotoxicity. The authors hypothesized that this result was due to the multifunctional design of the nanoplatform, including the tumor-targeting ability of HA, the mitochondrial-targeting capability of the TPP group, and the ROS-responsive cleavability of the thioester (TK) bond, which collectively enhance the photodynamic, gas, and ferroptotic therapeutic effects.

Given that GSH and GPX4 are key markers of ferroptosis, the authors measured their concentrations after treatment. The results showed that the HA@MR@PCN-CORM-treated group had the lowest levels of GSH and GPX4 (Figures 7F,G). Mechanistic validation also indicated that the HA@MR@PCN-CORM nanoplatform inhibited the activity of GCL and GS, key participants in GSH biosynthesis (Figure 7G). After elucidating the mechanism of tumor cell proliferation inhibition, the authors further validated the antitumor effects in animal models. The results demonstrated that, compared to various control groups, the HA@MR@PCN-CORM nanoplatform exhibited the strongest 4T1 cancer cell-killing effect (Figure 7H). In this system, the authors achieved a multifunctionally enhanced antitumor mechanism through the clever design of Por-nMOFs. Targeting mitochondria endowed the nanoplatform with more precise and synergistic antitumor effects. The successful design of this system provides new insights and references for the specific and functional design of Por-nMOFs.

**FIGURE 8:**
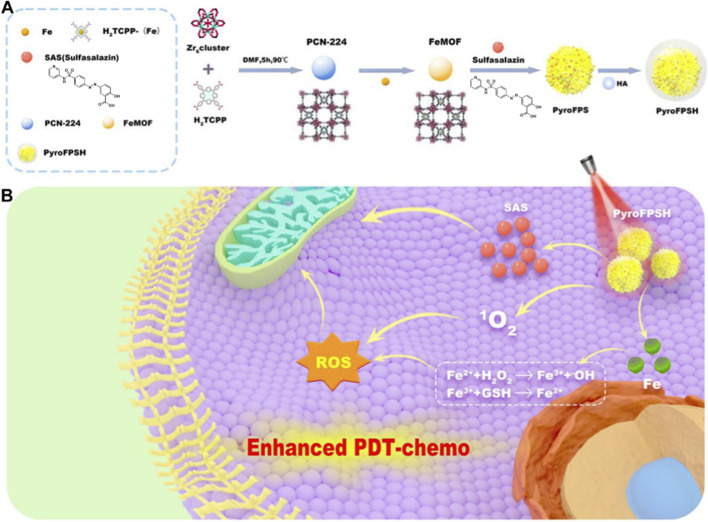
**(A)** Synthesis process of PyroFPSH, and **(B)** its tumor inhibition application. Adapted with permission from Lv et al. (2024). licensed under CC BY 4.0, Frontiers Media SA.

The “PDT + gas therapy” strategy has opened a new avenue for synergistic cancer treatment (Xie et al., 2025; Zhang S. et al., 2025; Gao et al., 2026; Peng et al., 2026). Likewise, leveraging the intrinsic multi-enzyme-like activities of nanomaterials to disrupt cellular redox homeostasis represents another powerful approach for inducing potent cytotoxicity. For example, Yang et al. reported a nanozyme-functionalized MOF (termed PyroFPSH) that exhibits glutathione peroxidase- and catalase-mimicking activities. This system effectively depletes intracellular glutathione, generates ROS, and co-delivers a mitochondria-depolarizing agent, thereby overcoming apoptosis resistance in cancer cells. Firstly, They synthesized PCN-224 MOFs using a conventional solvothermal method and loaded them with Fe elements and the small molecule sulfasalazine (SAS) *via* the MOF’s pores (Lv et al., 2024). The surface of the MOFs was then modified with HA, resulting in the formation of the HA@SAS@FeMOF (PyroFPSH) nanoplatform (Figure 8A) (Lv et al., 2024). Subsequent experimental results demonstrated that PyroFPSH, upon entering the cells, releases Fe elements. Fe^3+^ ions can react with intracellular GSH, reducing GSH concentrations and forming Fe^2+^ ions. These Fe^2+^ ions can further react with H_2_O_2_ in the cells, generating highly reactive OH. Additionally, the released SAS molecules target the mitochondria, causing mitochondrial depolarization, which prevents further O_2_ consumption and energy production for cellular metabolism. Thus, the designed PyroFPSH system achieves a multifunctional and synergistically enhanced mechanism (Figure 8B). On one hand, SAS molecules reduce the consumption of O_2_ by mitochondria, while Fe^3+^ ions lower intracellular GSH levels, disrupting the cellular redox balance. On the other hand, light-activated porphyrins generate ^1^O_2_, and Fe^2+^ ions react with H_2_O_2_ to produce OH. The combination of these mechanisms makes PyroFPSH a highly effective mitochondria-targeting photosensitizing nanoplatform.

**FIGURE 9:**
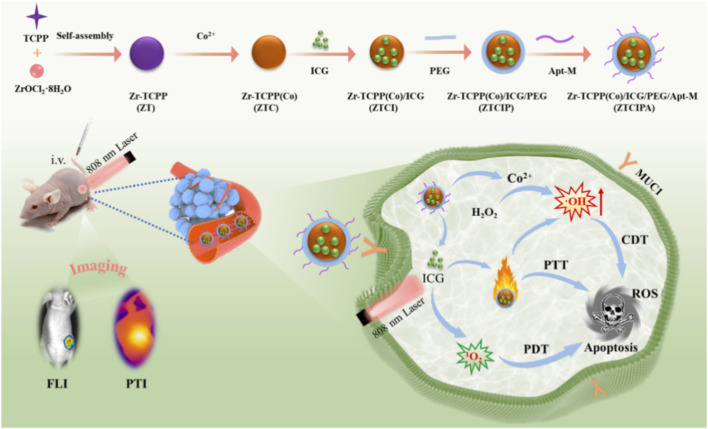
Fabrication procedure of the ZTCIPA nanoplatform and its underlying mechanisms for combined PTT, PDT, and CDT in antitumor applications. Adapted with permission from Zhao et al. (2025). © 2025 Elsevier B.V.

Multienzyme-mimetic nanoplatforms can simultaneously disrupt redox homeostasis through multiple mechanisms. Combining this “ROS storm” strategy with photothermal effects further amplifies mitochondrial damage. For instance, Feng et al. developed a Zr/Co-porphyrin-based MOF theranostic agent (denoted ZTCIPA) (Figure 9) (Zhao et al., 2025). The authors demonstrated that under 808 nm laser irradiation, ZTCIPA generates both photothermal heat and ^1^O_2_. Moreover, the localized hyperthermia accelerates Co^2+^-mediated catalysis of endogenous hydrogen peroxide in cancer cells, producing highly reactive OH. Together with ^1^O_2_, these species orchestrate a potent “ROS storm” that severely damages mitochondria, leading to effective suppression of cancer cell proliferation.

**FIGURE 10:**
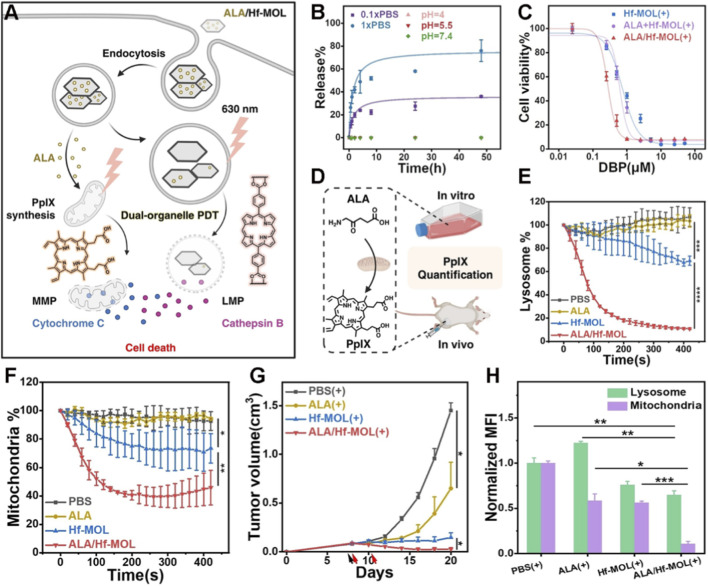
**(A)** Schematic illustration of the mechanism by which ALA/Hf-MOL targets mitochondria and lysosomes to suppress cancer cell proliferation. **(B)** Release profile of ALA under different pH conditions. **(C)** Viability of cancer cells treated under three different conditions as a function of DBP concentration. **(D)** Mechanism of ALA conversion to protoporphyrin IX (PpIX) under *in vitro* and *in vivo* conditions. **(E,F)** Fluorescence intensity changes in lysosomes **(E)** and mitochondria **(F)** following treatment with different experimental groups. **(G)** Tumor volume changes after treatment with different experimental groups. **(H)** Quantification of viable mitochondria and lysosomes following treatment with different experimental groups. Adapted with permission from Luo et al. (2023). licensed under CC BY 4.0, Wiley-VCH GmbH.

The “ROS storm” strategy has dramatically enhanced therapeutic lethality. Beyond leveraging exogenous effector molecules, optimizing the intracellular spatial distribution of photosensitizers to simultaneously attack multiple organelles can also induce lethal synergistic effects. For example, Luo et al. developed a dual-organelle–targeting platform (ALA/Hf-MOL) that concurrently targets mitochondria and lysosomes (Luo et al., 2023). This system enables *in situ* synthesis of protoporphyrin IX (PpIX) from 5-aminolevulinic acid (ALA) within mitochondria, while the MOF-based photosensitizer is retained in lysosomes. Upon light irradiation, both organelles are synchronously damaged, resulting in synergistically amplified PDT efficacy (Figure 10A). The authors found that ALA release from the platform was pH-independent but significantly enhanced with increasing phosphate concentration (Figure 10B). Although ALA/Hf-MOL and Hf-MOL exhibited comparable ROS generation capacities, phototoxicity assays under 630 nm irradiation revealed that the IC_50_ value of the ALA/Hf-MOL group was 2.7-fold lower than that of the Hf-MOL group, demonstrating markedly improved phototherapeutic efficacy after ALA loading (Figure 10C). This enhancement stems from the intracellular conversion of ALA into PpIX, which itself acts as an additional photosensitizer (Figure 10D). To validate the dual-organelle targeting effect, the authors examined mitochondrial and lysosomal depolarization. As shown in Figures 10E,F, the ALA/Hf-MOL group exhibited the most pronounced decrease in organelle-specific fluorescence just 2 min post-irradiation compared to all control groups, confirming its superior capacity to disrupt both mitochondria and lysosomes. Subsequent *in vivo* experiments further demonstrated that the ALA/Hf-MOL group achieved the strongest tumor growth suppression (Figure 10G). Additionally, co-staining of intratumoral mitochondria and lysosomes revealed the most significant reduction in both organelles in the ALA/Hf-MOL-treated group (Figure 10H). By simultaneously compromising two critical organelles, lysosomes and mitochondria, this system achieves highly effective inhibition of cancer cell proliferation through synergistic action. This work provides valuable preclinical insights for the development and application of porphyrin-based MOF materials in cancer therapy.

## 4 Por-nMOFs for enhanced SDT

The successful application of mitochondrial targeting in PDT has inspired its extension to other ROS-based anticancer modalities. SDT, which leverages the deep tissue penetration of ultrasound to activate sonosensitizers and generate ROS, depends on ultrasound-triggered cavitation. This process involves microbubble dynamics that produce local extreme conditions, activating sonosensitizers and inducing ROS generation directly. It has emerged as a powerful complementary approach for treating deep-seated or occult tumors (Chen et al., 2024; Das et al., 2024; Liu et al., 2024; Wang et al., 2024). Similar to PDT, SDT faces significant challenges, including robust tumor antioxidant defenses and limited targeting efficiency of sonosensitizers. To address these limitations, Por-nMOFs have been explored as sonosensitizer carriers, integrated with mitochondrial targeting and microenvironment-modulating functionalities to enhance SDT efficacy.

**FIGURE 11:**
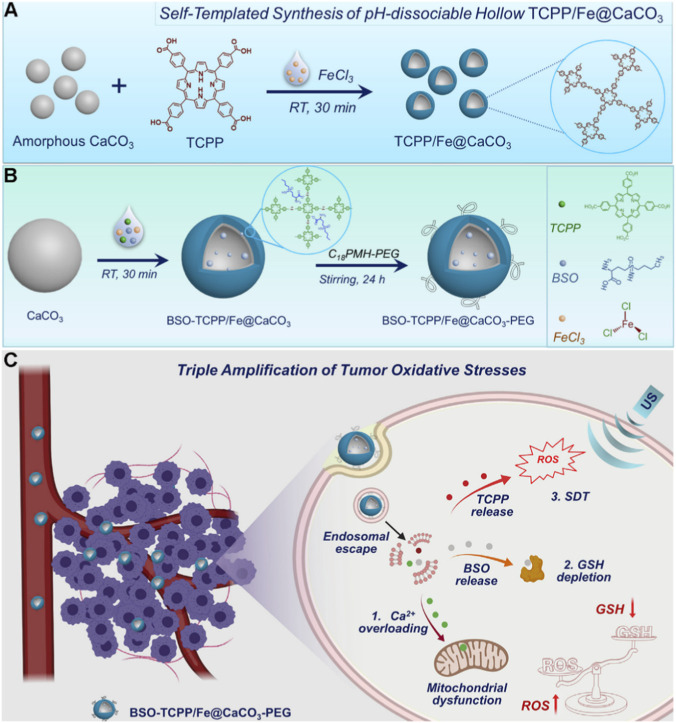
**(A)** Schematic illustration of the synthesis procedure for the TCPP/Fe@CaCO_3_ composite. **(B)** Design rationale of the BSO-TCPP/Fe@CaCO_3_-PEG nanocomposite. **(C)** Schematic representation of the synergistic chemotherapeutic and SDT-mediated inhibition of cancer cell proliferation by BSO-TCPP/Fe@CaCO_3_-PEG. Adapted with permission from Dong et al. (2020). © 2020 Elsevier Inc.

For instance, Dong et al. developed a novel pH-responsive hollow coordination architecture, TCPP/Fe@CaCO_3_ (Figure 11A) (Dong et al., 2020). During synthesis, the GSH synthesis inhibitor buthionine sulfoximine (BSO) was co-loaded, yielding the multifunctional nanocomposite BSO-TCPP/Fe@CaCO_3_-PEG (Figure 11B). The authors demonstrated that this system responds specifically to the acidic tumor microenvironment, triggering the simultaneous release of BSO and Ca^2+^ ions. BSO suppresses intracellular GSH biosynthesis, weakening the antioxidant capacity of cancer cells, while Ca^2+^ overload disrupts mitochondrial function. This dual action, chemosensitization *via* Ca^2+^-induced mitochondrial dysfunction and GSH depletion, synergizes with ultrasound-triggered ROS generation from the TCPP sonosensitizer, creating a multi-pronged amplification of oxidative stress that directly eradicates tumor cells (Figure 11C). This elegantly designed yet straightforward platform exemplifies how strategic targeting of mitochondrial integrity, combined with rapid responsiveness to the tumor microenvironment, can significantly potentiate SDT mediated by Por-nMOFs, offering a promising new paradigm for enhanced sonodynamic cancer therapy.

**FIGURE 12:**
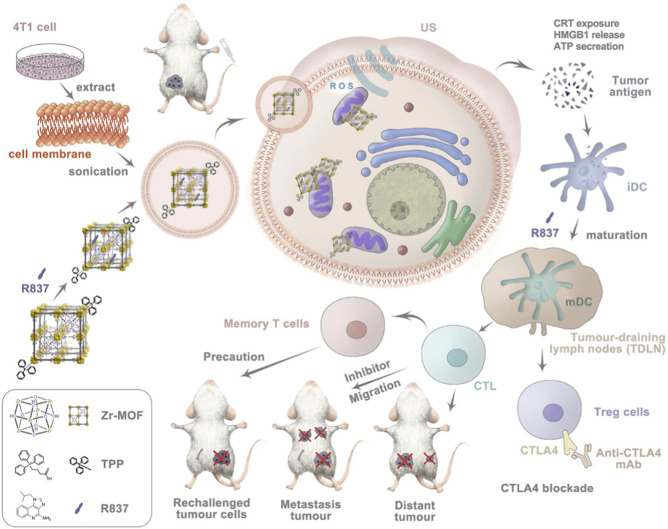
Schematic illustration of Zr-TCPP(TPP)/R837@M exerting combined SDT and immunogenic cell death (ICD) effects to suppress cancer cell proliferation. Adapted with permission from Luo et al. (2022). licensed under CC BY 4.0, Springer Nature.

Strategies that amplify SDT efficacy by disrupting intrinsic cellular homeostasis offer promising avenues to overcome tumor resistance. However, beyond direct tumor cell killing, eliciting robust antitumor immune responses, capable of achieving long-term control and immunological memory, represents an even more compelling therapeutic direction. For example, Huang et al. engineered a biomimetic nanoplatform, Zr-TCPP(TPP)/R837@M, by coating a TPP-functionalized porphyrin-based MOF with cancer cell membranes (Luo et al., 2022). This design integrates homotypic tumor targeting (conferred by the cancer cell membrane cloak) with mitochondrial targeting (TPP) (Figure 12). The authors demonstrated that, upon ultrasound activation, the sonosensitizer TCPP not only exerts potent SDT effects but also significantly enhances immunogenic cell death (ICD). The resulting exposure of damage-associated molecular patterns (DAMPs) promotes dendritic cell maturation and T-cell priming. Furthermore, co-delivery of the Toll-like receptor 7 agonist R837 synergizes with SDT-induced ICD to reprogram the immunosuppressive tumor microenvironment. This combination effectively bridges innate and adaptive immunity and enables potent synergy with immune checkpoint blockade therapy, thereby establishing a robust SDT-immunotherapy alliance.

Although Por-nMOF-facilitated SDT has demonstrated efficacy in surmounting the tissue penetration barrier of conventional PDT, it still encounters bottlenecks in the treatment of deep-seated tumors or highly drug-resistant malignancies. This clinical need drives the exploration of energy activation modalities with superior tissue penetrability, thus promoting the advancement toward RDT integrated with radiotherapy (RT). It is noteworthy that two core issues dominate clinical translatability. First, the refinement of RT dosing regimens: consensus is lacking regarding the optimal fractional dose and cumulative dose for distinct tumor subtypes, and the therapeutic benefit of dose escalation without exacerbating normal tissue toxicity awaits validation in large-scale clinical investigations. Second, the precision improvement of RT target volume delineation: accurate identification of tumor margins and hypoxic foci remains a clinical hurdle; ongoing trials integrating functional imaging modalities with RT target planning strive to enhance radiation delivery precision and mitigate off-target adverse effects. These unresolved clinical challenges underscore the imperative for innovative radiosensitizing strategies, which extends the therapeutic utility of Por-nMOFs to refractory deep-seated tumors.

## 5 Por-nMOFs for enhanced RDT

RT, or more specifically RDT, utilizes high-energy ionizing radiation to directly damage tumor cells and can be locally amplified by radiosensitizers, particularly high-Z elements such as Hf. The radio-sensitization mechanism in RDT primarily involves high-Z elements enhancing the capture of ionizing radiation, which amplifies local energy deposition and promotes the generation of ROS and free radicals. These reactive species further induce oxidative damage to tumor cell DNA, exacerbating radiation-induced cell death. Integrating such radiosensitizing metals into the framework of Por-nMOFs, while further incorporating radiation-triggered therapeutic modules within a mitochondria-targeting architecture, represents a strategic evolution from PDT and SDT toward RDT. This shift in energy modality not only extends the utility of Por-nMOFs beyond superficial tumors but also opens new avenues for eradicating refractory deep-seated malignancies.

**FIGURE 13:**
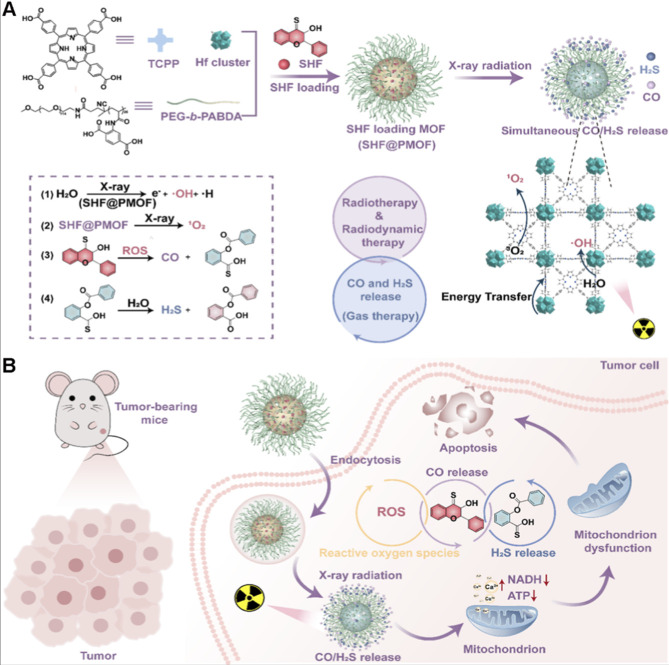
**(A)** Schematic illustration of the fabrication of the SHF@PMOF nanoplatform and the mechanism of X-ray-triggered co-release of carbon monoxide (CO) and hydrogen sulfide (H_2_S). **(B)** Schematic representation of the multifunctional SHF@PMOF nanoplatform for inhibiting cancer cell proliferation. Adapted with permission from Cao et al. (2025). Copyright © 2025 American Chemical Society.

For instance, Ge et al. developed a multifunctional polymer-metal-organic framework (PMOF)-based nanoplatform, termed SHF@PMOF (Figure 13A) (Cao et al., 2025). This system integrates X-ray-triggered dual-gas (CO/H_2_S) release with potent radiosensitization to significantly enhance RT efficacy. The PMOF scaffold is constructed from the high-Z element Hf and the photosensitizing ligand TCPP, forming a porous structure that (i) maximizes X-ray absorption and secondary electron emission, thereby boosting the generation of ·OH and ^1^O_2_, and (ii) enables stable encapsulation and controlled release of the dual-gas donor SHF. Unlike PDT, limited by shallow light penetration, or SDT, which suffers from relatively low sonosensitizer-to-ROS conversion efficiency despite improved depth access, the RT/RDT strategy leverages X-rays’ exceptional tissue penetration and strong ionizing capacity. This allows simultaneous activation of ROS production and gas release deep within tumors, effectively overcoming the physical constraints of PDT and SDT. Mechanistically, the co-released CO and H_2_S synergistically disrupt mitochondrial function by inhibiting ATP synthesis, perturbing Ca^2+^ homeostasis, and suppressing NADH dehydrogenase activity, thereby markedly increasing tumor cell radiosensitivity (Figure 13B). Both *in vitro* and *in vivo* experiments confirmed that SHF@PMOF achieves potent antitumor effects under low-dose X-ray irradiation while minimizing collateral damage to healthy tissues. Notably, this work represents the first application of a PMOF platform for the co-delivery of a dual-gas donor and a radiosensitizer, establishing a novel “RT-RDT + gas therapy” synergistic paradigm. It provides a promising blueprint for overcoming tumor radioresistance and expands the therapeutic arsenal against deep and treatment-refractory cancers.

## 6 Conclusion and perspectives

### 6.1 Summary of mitochondrial-targeted Por-nMOFs in antitumor therapies

This review systematically explores Por-nMOFs as multifunctional nanoplatforms for enhancing synergistic antitumor strategies in PDT, SDT, and RDT through mitochondrial targeting. These Por-nMOFs, owing to their high porosity, tunable chemical structures, and intrinsic photosensitizing properties, serve as ideal carriers or active components for photo/sono/radio-sensitizers. By modifying with mitochondrial-targeting ligands such as triphenylphosphonium, these nanoplatforms can specifically accumulate in the mitochondria of tumor cells, the cellular powerhouses closely linked to ROS-mediated cell death pathways. In PDT, under NIR or visible light excitation, MOFs generate ^1^O_2_, and mitochondrial targeting not only increases the local ROS concentration at the subcellular level but also alleviates tumor hypoxia, a major bottleneck of PDT, by interfering with the respiratory chain or producing O_2_, thereby significantly amplifying therapeutic efficacy. In SDT, ultrasound-activated porphyrin MOFs also produce ^1^O_2_, and combining mitochondrial targeting with strategies like calcium overload or GSH depletion synergistically amplifies oxidative stress, enhancing sonodynamic killing efficiency. In RDT, the incorporation of high atomic number metals enables porphyrin MOFs to efficiently absorb X-rays, generating ·OH and ^1^O_2_ *via* the RT-RDT mechanism; meanwhile, loading dual gas donors and triggering their release under X-ray irradiation can induce mitochondrial dysfunction, radiosensitizing tumors and achieving synergy between gas therapy and RDT. In summary, Por-nMOFs, through the precise integration of mitochondrial targeting with multiple energy excitation modes (light, ultrasound, X-rays) and auxiliary strategies (hypoxia modulation, oxidative stress amplification, gas therapy), construct efficient, multi-layered nanoplatforms for tumor treatment, demonstrating significant potential for clinical translation.

### 6.2 Challenges in clinical translation

Despite the notable advantages of Por-nMOFs in enhancing PDT, SDT, and RDT *via* mitochondrial targeting, their further development and clinical translation face several challenges. First, the contradiction between physiological stability and biodegradability is prominent: Notably, MOF biodegradation is often accompanied by metal ion release (*e.g.*, Hf^4+^, Zr^4+^), which can induce oxidative stress, inflammatory responses, or organ damage through long-term accumulation *in vivo*, posing a major obstacle to biocompatibility. Although coatings like ZIF-8 or polymer modifications can improve stability, they may affect drug loading/release kinetics or biodegradability. Second, balancing targeting efficiency and systemic toxicity requires optimization: cationic targeting ligands like TPP, while effective for mitochondrial accumulation, can cause nonspecific adsorption, aggregation, and rapid clearance in systemic circulation, potentially inducing toxicity to normal cell mitochondria. Moreover, TPP may exhibit off-target mitochondrial effects towards some normal cells (e.g., cardiomyocytes, neurons) with high membrane potential, leading to unintended accumulation and disruption of normal mitochondrial function, which may trigger cardiotoxicity or neurotoxicity. Existing “stealth” strategies (hyaluronic acid coating) improve pharmacokinetics but still need enhancement in triggered exposure efficiency within the tumor microenvironment. In terms of pharmacokinetics and long-term clearance, current Por-nMOF systems often suffer from poor blood circulation stability and rapid reticuloendothelial system clearance, resulting in low tumor accumulation efficiency. Meanwhile, the long-term clearance pathways of MOF degradation products, including metal ions and ligand fragments, remain unclear, and their potential long-term biological effects have not been fully evaluated.

In addition, therapeutic efficacy is limited by the complexity of the tumor microenvironment: although O_2_-generating nanozymes or respiratory inhibitors are integrated to alleviate hypoxia, insufficient H_2_O_2_ supply in tumors, pH-dependent catalytic efficiency (MnO_2_ deactivation in acidic environments), and metabolic heterogeneity among cell types may lead to unstable O_2_ enhancement effects. Furthermore, the construction and regulation of multimodal synergistic therapies remain complex: integrating MOFs with UCNPs, CDs, or other nanozymes often involves complicated synthesis steps, poor structural homogeneity, and insufficient energy transfer efficiency; additionally, precise spatiotemporal control over the release sequence of multiple therapeutic components to maximize synergy is still poorly understood. Finally, research on the impact on the immune microenvironment and long-term antitumor mechanisms is insufficient: current studies focus primarily on direct cell killing, while the ICD induced by porphyrin MOF-mediated PDT/SDT/RDT and its synergistic potential with immunotherapy remains underexplored, limiting their ability to combat metastasis and recurrence.

### 6.3 Future research directions

To address these challenges, future research should advance from material design, biomedical engineering, to clinical translation. Regarding stability and biocompatibility, novel “smart” coatings or hybrid structures can be developed: for example, designing pH- or enzyme-responsive polymer shells that remain stable in circulation but degrade at the tumor site to expose targeting ligands, enabling precise delivery; simultaneously, exploring more stable MOF materials or developing self-sacrificial coatings (ZIF-8) could balance stability with controlled release. For mitigating metal ion release toxicity, surface modification with chelating ligands or incorporation of metal ion scavenging components into MOF frameworks can be considered to reduce free metal-ion accumulation *in vivo*. To enhance targeting specificity and safety, multi-stage targeting strategies should be pursued: for instance, co-modifying MOF surfaces with tumor-targeting molecules and organelle-targeting ligands to enhance tumor accumulation *via* EPR effect and active targeting, followed by improved subcellular localization through mitochondrial targeting; moreover, developing new mitochondrial-targeting groups or utilizing mitochondrial-specific signals (membrane potential) to trigger drug release may reduce systemic toxicity. To optimize long-term clearance and pharmacokinetics, modifying MOFs with hydrophilic, non-immunogenic polymers (*e.g.*, PEG derivatives, zwitterionic polymers) can extend blood circulation time. Meanwhile, designing MOFs with biodegradable backbones that can be completely degraded into metabolizable small molecules and non-toxic metal ions will promote long-term clearance and reduce chronic toxicity.

For optimizing tumor microenvironment modulation, more efficient catalytic systems need to be designed: for example, constructing bimetallic MOFs (Zr/Co, Fe/Cu) to synergistically enhance Fenton-like reactions and photocatalytic performance, or developing self-O_2_-supplying and self-amplifying ROS-generating nanozymes for sustained H_2_O_2_ and O_2_ supply; concurrently, combining metabolic regulation (simultaneous inhibition of glycolysis and oxidative phosphorylation) or calcium overload strategies could dismantle tumor defenses through multiple pathways. To simplify multifunctional integration and precise control, “all-in-one” MOF designs should be advanced: for example, embedding upconversion CDs, nanozymes, and targeting ligands directly into the MOF or pores *via* one-pot synthesis to shorten synthetic routes and ensure uniform dispersion; leveraging the spatiotemporal differences of external stimuli (light, ultrasound, X-rays) to program the sequential activation of different therapeutic modes, such as triggering PDT with NIR light to generate ROS first, followed by X-ray-activated gas release to enhance RDT. Finally, research on immune synergistic therapy and long-term efficacy should be expanded: co-loading porphyrin MOFs with immune adjuvants and combining them with immune checkpoint inhibitors (anti-CTLA-4 antibodies) can induce ICD through SDT/PDT to release tumor antigens, simultaneously activating dendritic cells and T cells to establish systemic antitumor immune memory; additionally, utilizing the modifiability of MOFs to load reporter genes or contrast agents could enable real-time imaging monitoring of the treatment process, promoting theranostics.

### 6.4 Clinical translation outlook

For the clinical translation of Por-nMOFs, three key issues need to be solved: safety, scalability, and dosing. In terms of safety, a comprehensive preclinical system should be established, including acute/chronic toxicity tests, long-term organ function monitoring, and immunogenicity evaluations. Special attention should be paid to the potential off-target effects of TPP and metal ion release toxicity, and the corresponding mitigation strategies (chelation modification, targeted ligand optimization, *etc.*) must be verified in large animal models. Regarding scalability, current laboratory-scale synthesis methods are difficult to meet clinical demand. Therefore, developing scalable synthesis technologies with low cost and good reproducibility is essential. In terms of dosing, personalized dosing methods considering patient characteristics, tumor type, and size should be developed. Moreover, the pharmacokinetic properties of Por-nMOFs in different patients need to be clarified to avoid adverse reactions induced by individual differences. Additionally, as novel nanomedicines, Por-nMOFs must comply with regulatory medical guidelines and criteria for safety and efficacy to accelerate the clinical translation process. In conclusion, with the integration of materials science, nanotechnology, and tumor biology, Por-nMOFs are poised to evolve into intelligent, personalized, multimodal synergistic platforms for tumor therapy, ultimately achieving clinical translation.
